# The GP tests of competence assessment: which part best predicts fitness to practise decisions?

**DOI:** 10.1186/s12909-017-1111-0

**Published:** 2018-01-02

**Authors:** Hirosha Keshani Jayaweera, Henry W. W. Potts, Karim Keshwani, Chris Valerio, Magdalen Baker, Leila Mehdizadeh, Alison Sturrock

**Affiliations:** 1 Research Department of Medical Education, University College London, London, UK; 2 Institute of Health Informatics, University College London, London, UK

**Keywords:** Tests of competence, General practice, OSCEs, Simulated surgery

## Abstract

**Background:**

The General Medical Council (GMC) conducts Tests of Competence (ToC) for doctors referred for Fitness to Practise (FtP) issues. GPs take a single best answer knowledge test, an Objective Structured Clinical Examination (OSCE), and a Simulated Surgery (SimSurg) assessment which is a simulated GP consultation. The aim of this study was to examine the similarities between OSCEs and SimSurg to determine whether each assessment contributed something unique to GP ToCs.

**Methods:**

A mixed methods approach was used. Data were collated on 153 GPs who were required to undertake a ToC as a part of being investigated for FtP issues between February 2010 and October 2016. Using correlation analysis, we examined to what degree performance on the knowledge test, OSCE, and SimSurg related to case examiner recommendations and FtP outcomes, including the unique predictive power of these three assessments. The outcome measures were case examiner recommendations (i) not fit to practise; ii) fit to practise on a limited basis; or iii) fit to practise) as well as FtP outcomes (i) erased/removed from the register; ii) having restrictions/conditions; or iii) be in good standing).

For the qualitative component, 45 GP assessors were asked to rate whether they assess the same competencies and which assessment provides better feedback about candidates.

**Results:**

There was significant overlap between OSCEs and SimSurg, *p* < 0.001. SimSurg had additional predictive power in the presence of OSCEs and the knowledge test (*p* = 0.030) in distinguishing doctors from different FtP categories, while OSCEs did not (*p* = 0.080). Both the OSCEs (*p* = 0.004) and SimSurg (*p* < 0.001) had significant negative correlations with case examiner recommendations when accounting for the effects of the other two assessments.

Inductive thematic analysis of the responses to the questionnaire showed that assessors perceived OSCEs to be better suited to target specific knowledge and skills. SimSurg was thought to produce a more global picture as the scenarios more accurately portray a patient consultation.

**Conclusion:**

While all three assessments are strong predictors of both case examiner recommendations and FtP outcomes, our findings suggest that the efficiency of GP ToCs can be improved by removing some of this overlapping content.

## Background

The General Medical Council (GMC) holds the right to assess the fitness to practise (FtP) of doctors on the medical register in the UK [1]. In 2015, the GMC received 9092 enquiries about doctors, of which 91% concerned FtP issues. The public made 68% of these complaints, 9% were made by other doctors, 6% by employers, and 17% from other sources [2]. After these complaints are received, the GMC triage them and decide whether or not to launch a full investigation [3]. Allegations about FtP are broadly categorised into health, conduct and/or performance related issues [2].

Part of the GMC investigation into doctors who are referred for fitness to practise following concerns around their performance at work may include a Test of Competence (ToC) [4]. The ToC is used to identify potential gaps in a doctor’s knowledge base and/or clinical skills [1]. For the majority of doctors, this test comprises a written knowledge test with 120 questions in the Single Best Answer (SBA) format, and a 12 station Objective Structured Clinical Exam (OSCE). The items included in a ToC have all been tested on groups of volunteer doctors through pilot events organised by University College London (UCL) Medical School. During these events, the items are tested to ensure they are fair and fit for purpose for that specialty. The actual assessments are tailored to the individual doctor’s grade, specialty, and area of clinical work [5]. There is no minimum ‘pass mark’ for these assessments; marks obtained by the doctor under investigation are compared to the range of marks obtained by a group of volunteer doctors in a similar test in the same specialty [3, 5].

The results of the ToC assessments are used alongside the results obtained from a peer review exercise to help the assessment team write a report that includes a recommendation about the doctor. This recommendation is then reviewed by two case examiners (one medical, one non-medical) who can conclude the case with no further action, issue a warning, agree undertakings with the doctor, or refer the case to the Medical Practitioners Tribunal Service (MPTS) for a hearing [6].

MPTS is a statutory committee of the GMC that has independent decision-making authority, and is responsible for adjudicating FtP cases [6]. The tribunal consists of three members, of whom at least one is a doctor, and one is a lay person. It is chaired by a legally qualified member. The tribunal members hear the evidence provided by the GMC, and conclude the hearing with one of several outcomes. These outcomes are: the doctor remaining on the medical register *without* warnings or conditions, the doctor remaining on the register with warnings/conditions, or the doctor being erased from the register [7].

**Fig. 1:**
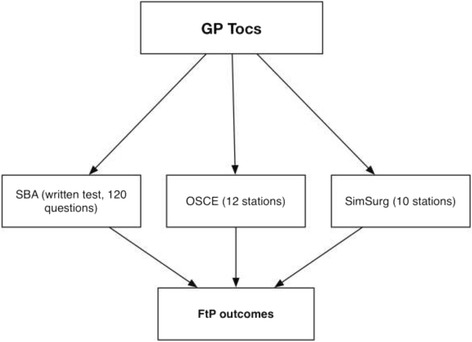
Schematic representation of the assessments included in the GP tests of competence (ToCs). Performance in these three assessments then contribute to Fitness to Practise (FtP) final outcomes

General practice is the specialty that receives most complaints: they were involved in 43% of all complaints in 2015 [2], accounting for 5% of all registered General Practitioners (GPs). In addition to a knowledge test and OSCEs, GPs also take a tailored Simulated Surgery (SimSurg) assessment that consists of ten stations, each a simulated patient consultation with a presenting complaint [8]. The sequence of assessments completed by GPs is presented in Fig. 1. In contrast to an OSCE station, the doctor undergoing assessment stays in the room without moving from one station to the next, and the examiner is able to view the consultation via video-link. While SimSurg has been devised to reflect how GPs work in practise, there are similarities between the scenarios used in SimSurg and some of the scenarios used in OSCE stations. This raises the question of whether it is necessary to include both an OSCE and a SimSurg in a GP ToC assessment. Reducing the amount of assessment would reduce the amount of stress for the doctor being investigated, as well as the cost and workload for the assessors. Further, in contrast to the use of both SimSurg and OSCEs in GP ToCs, the MRCGP examination’s Clinical Skills Assessment (CSA) only contains one type of clinical assessment, which involves realistic simulation of real-life consultation stations, much like the SimSurg component of the GP ToCs [9]. Hence, having only one form of clinical skills assessment would bring GP ToCs more in line with the format of the CSA component of the MRCGP examination.

The overall aim of this study was to examine the similarities between OSCEs and SimSurg to quantitatively determine whether each assessment contributed something unique to GP ToCs, and to assess the perceived benefits and limitations of both modalities from a qualitative perspective.

Specific objectives:

1. Examine the relationship between each assessment and FtP outcomes/case examiner recommendations.
2. Examine the correlations between FtP outcomes and case examiner recommendations.
3. Examine the unique predictive power of each assessment in terms of FtP outcomes and case examiner recommendations.
4. Explore assessors’ opinions of OSCEs and SimSurg and how well these correspond to the quantitative findings.

## Method

Mixed method research methodology involves the integration of both qualitative and quantitative research designs [10]. We took a mixed methods approach for this study to provide a quantitative (statistical) analysis of GP ToCs assessments whilst also providing detailed examples of individual assessor’s opinions of the assessments.

### Participants

We examined quantitative data from doctors undergoing GP ToCs conducted between February 2010 and October 2016, identified through the GMC register of doctors that have been investigated for fitness to practise purposes. For the qualitative component, the GMC training team contacted all of the 45 assessors that had performed a GP assessment in the past 18 months. They were sent an email with a link to the questionnaire. The email explained ‘the aim of the study is to gather your views about these assessment methods in order to ensure our GP ToC assessments are efficient and remain of high standards’.

### Data collection

We examined the ability of each assessment to predict case examiner recommendations and FtP outcomes. The possible case examiner recommendations were:

i) not fit to practise;
ii) fit to practise on a limited basis;
iii) fit to practise.

The possible FTP outcomes were:

i) erased/removed from the register (including those that relinquished their medical licence during the FtP process as well as those that were erased at the end of the FtP process);
ii) having restrictions/conditions on the GMC registration;
iii) or be in good standing without conditions.

For the qualitative component, we asked GP assessors who were involved in conducting ToCs between September 2014 and March 2016 about the utility of OSCEs and SimSurg in assessing GP’s fitness to practice.

1. **Performance assessment**

The 120 item SBA knowledge test chosen specifically for each doctor under investigation was computer marked, with a mark out of 120 converted to a percentage for reporting. The 12 bespoke OSCE stations chosen for each doctor were marked out of 40, as well as using the categories ‘acceptable’ ‘cause for concern’ and ‘unacceptable’. The 10 SimSurg assessments prior to October 2014 were in the form of a set roster of stations of which there were two rosters (first sit and resit assessment), after October 2014, this was changed to 10 bespoke stations. Due to these differences, and the bespoke nature of most assessments, only total scores in each assessment as a percentage of the total score has been used in the data analysis.

We performed between-subjects ANOVA to look for group differences. We used Pearson’s r correlation to examine the overlap between the assessments with each assessment as a predictor where FtP outcome and case examiner recommendation were dependent variables. We used Spearman’s correlation to examine the overlap in the FtP outcome categories and case examiner recommendation. Fisher’s transformations were conducted to examine the unique contribution of each assessment to FtP outcomes and case examiner recommendations. All analyses were conducted using SPSS version 22.

2) **Assessors’ opinions**

AS, KK, CV, LM developed a short 10–15 min survey using UCL’s secure online questionnaire platform, Opinio. The survey ran from 23 March to 19 May 2016. All responses were anonymous. The questions were formulated through team discussion and the final version was reviewed and approved by AS, KK, CV, and LM. Respondents were asked to express views regarding OSCE and SimSurg in free text box responses. The questions posed for this free text responses were: *“Are there benefits of using OSCEs over SimSurg?*” and *“Are there benefits of using SimSurg over OSCEs*”. We also asked respondents to rate agreement with statements about whether the two modalities assess the same competencies, and which provides better feedback about candidates’ performance. We performed inductive thematic analysis on the free text responses based on the method described by Braun and Clark [11]. Specifically, two researchers (KK and CV) reviewed to survey responses and developed a coding framework. Surveys were then divided evenly between these two researchers and coded independently. This was followed by a discussion of areas of uncertainty and reviewing of the themes.

## Results

Overall, 153 GPs took a ToC between February 2010 and October 2016. For the qualitative component, 34 of the 45 GP assessors responded, giving a response rate of 76%.

### Performance assessment

#### Demographics

The demographics of the GPs are summarised in Table 1. GPs that were investigated for FtP issues were more likely to be men and international medical graduates. While the year of primary medical qualification ranged from 1961 to 2007, the majority of doctors qualified before 1992.

**Table 1:** Demographics of doctors under FtP investigation between February 2010 and October 2016

Category	Number of doctors
Sex	
Female	30
Male	123
Qualification	
United Kingdom	64
European Economic Area	18
International	71
PMQ year	
1961–1992	127
1993–2005	25
2006–2016	1

##### Test of competence score in relation to final FtP outcome

In terms of the final FtP outcomes, 63 of the 153 GPs were no longer on the GMC register (including 42 that relinquished their medical licence during the FtP process), 50 were on the register with warnings, conditions, suspensions, and/or undertakings, while 40 were on the register in good standing.

Average scores on the SBA knowledge test, OSCEs and SimSurg for GPs in each of the three outcome groups are shown in Table 2. There were overall significant group differences in the assessment scores when examining doctors from different outcome categories for all three assessments (Table 2).

**Table 2:** Average scores on the knowledge test, OSCEs and SimSurg for each FtP outcome category

	On the register in good standing (group 1, *N* = 40)	On the register with warnings/sanctions (group 2, *N* = 50)	No longer on the GMC register (group 3, *N* = 63)	Significance
Knowledge test score				
Mean	71.7	68.7	55.9	*F*(2150) = 24.8
Standard deviation	7.3	10.2	16.0	*p* < 0.001
OSCEs total score				
Mean	82.0	74.9	58.4	*F*(2150) = 29.6
Standard deviation	11.5	15.7	18.7	*p* < 0.001
SimSurg total score				
Mean	69.7	62.0	45.8	*F*(2150) = 28.6
Standard deviation	14.4	17.4	17.0	*p* < 0.001

Specifically, for the knowledge test score, OSCEs and SimSurg, post-hoc contrasts showed that GPs removed from the GMC register scored significantly lower than GPs with warnings/sanctions (*p* < 0.001), and GPs on the register in good standing (*p* < 0.001). There were no significant differences in any of the assessment scores when comparing GPs on the register with warnings/sanctions compared to GPs on the register in good standing (*p* > 0.05).

#### Correlation analysis

**Fig. 2:**
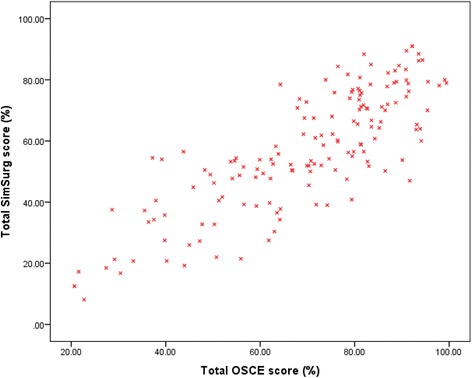
Scatterplot for the relationship between OSCEs and SimSurg

There were significant negative correlations between the outcomes of FtP ToCs and all the assessments (see Table 3). Fisher’s transformations demonstrated that as standalone assessments, all three assessments were equally strong at predicting FtP outcomes (*p* > 0.05). There were also significant positive correlations between the assessments with moderate to large effect sizes *p* < 0.001. As can be seen in Table 3, the largest overlap occurred between OSCEs and SimSurg (see Fig. 2), compared to the overlap between these assessments and the knowledge test.

**Table 3:** Correlations between assessments

	FtP outcome	Knowledge test	OSCEs
Knowledge test	−0.49 [−0.36, −0.60]		
OSCEs	−0.52 [−0.39, −0.63]	0.60 [0.49, 0.69]	
SimSurg	−0.53 [−0.41, −0.64]	0.62 [0.51, 0.71]	0.79 [0.72, 0.84]

Confidence intervals indicated in brackets. Pearson’s and Spearman’s correlations were used for the correlation between assessments, and correlation of assessments with FtP outcomes, respectively

Due to the significant correlation between the three assessment measures, partial correlations were conducted to examine the contribution of each assessment to FtP outcomes, while controlling for the effect of the other two assessments. There was a significant negative correlation between FtP outcomes and SimSurg, when accounting for the effect of OSCEs and the knowledge test (*ρ* = −0.17, *p* = 0.030). However, the relationship between OSCEs and FtP outcomes was no longer significant when accounting for the effect of SimSurg and the knowledge test (*ρ* = −0.14, *p* = 0.080). That is, while all three assessments were significant predictors of FtP outcomes, there was significant overlap in content, such that OSCEs did not provide additional predictive power when taking into account the effect of SimSurg and the knowledge test. In contrast, SimSurg did provide additional predictive power.

##### Test of competence score in relation to case examiner recommendations

In terms of the case examiner recommendations, 30 of the 153 GPs were recommended as fit to practise, 81 recommended as fit to practise on a limited basis, while 42 were recommended as not fit to practise.

Average scores for the SBA knowledge test, OSCEs and SimSurg for GPs in each of the three case examiner recommendation groups are shown in Table 4. There were overall significant group differences in the scores when examining doctors in the different case examiner recommendation categories for the knowledge test, OSCEs and SimSurg.

**Table 4:** Average scores on the knowledge test, OSCEs and SimSurg for each case examiner recommendation category

	Fit to practise (group A, *N* = 30)	Fit to practise on a limited basis (group B, *N* = 81)	Not fit to practise (group C, *N* = 42)	Overall group difference
Knowledge test score				
Mean	72.4	68.0	51.0	*F*(2150) = 39.7
Standard deviation	7.5	10.0	15.9	*p* < 0.001
OSCE total score				
Mean	86.2	74.4	49.8	*F*(2150) = 71.7
Standard deviation	9.4	12.6	17.5	*p* < 0.001
SimSurg total score				
Mean	75.4	61.1	37.3	*F*(2150) = 73.8
Standard deviation	11.6	14.2	14.3	*p* < 0.001

For all three assessments, post-hoc contrasts showed that GPs recommended as not fit to practise performed significantly poorer than GPs recommended as fit to practise on a limited basis (*p* < 0.001) and also compared to GPs recommended as fit to practise generally (*p* < 0.001). There were no significant differences in the knowledge test scores when comparing GPs recommended as fit to practise on a limited basis compared to those recommended as fit to practise generally (*p* > 0.05). However, GPs who were classified as fit to practise on a limited basis performed significantly lower on the OSCEs and SimSurg compared to those classified as fit to practise generally (*p* = 0.004 and *p* < 0.001, respectively).

#### Correlation analysis for case examiner recommendation categories

There were significant negative correlations between the case examiner recommendations and all the assessments, with moderate to large effect sizes, *p* < 0.001, see Table 5. Fisher’s transformations demonstrated that as standalone assessments, all three assessments were equally strong at predicting case examiner recommendations (*p* > 0.05).

**Table 5:** Correlations between assessments and case examiner recommendations

	Case examiner recommendation
Knowledge test	−0.55
OSCEs	−0.68
SimSurg	−0.70

Due to the significant overlap between the OSCEs and SimSurg, partial correlations examining the contribution of each assessment to case examiner recommendations, while controlling for the effects of the other assessments were conducted. There was a significant negative correlation between case examiner recommendations and SimSurg, when accounting for the effect of OSCEs and the knowledge test (*ρ* = −0.33, *p* < 0.001). There was also a smaller, significant negative correlation between case examiner recommendations and OSCEs, when accounting for the effect of SimSurg and the knowledge test (*ρ* = −0.23, *p* = 0.004).

##### Overlap between FtP outcomes and case examiner recommendations

A Spearman’s correlation examining the overlap between doctors in each FtP category and doctors in each case examiner recommendation category was statistically significant, demonstrating the large overlap between FtP outcome categories and case examiner recommendation categories, *ρ* = −0.64, *p* < 0.001 (see Table 6).

**Table 6:** Cross tabulation of the case examiner recommendations vs. final FtP outcomes

FtP outcome	Case examiner recommendation			
	Fit to practise (*N* = 30)	Fit to practise on a limited basis (*N* = 81)	Not fit to practise (*N* = 42)	Total
On the register (*N* = 40)	24	13	3	40
Warnings, conditions and undertakings (*N* = 50)	6	40	4	50
Erased from the register (*N* = 63)	0	28	35	63
Total	30	81	42	153

### Assessors’ opinions

#### Qualitative results

Overall, the inductive thematic analysis [10] of free text responses showed that assessors perceived OSCEs to be better suited to target specific knowledge and skills that SimSurg could not assess, such as colleague discussions. OSCEs were perceived to have lower face validity compared to SimSurg, whose scenarios more accurately portray a patient consultation. This “authenticity” was perceived to provide a more global picture of candidate performance. Specific skills, like prescribing, are assessed through integration within the consultation.

SimSurg was thought to be less intimidating because the assessor is not in the room, and did not penalise candidates who were not accustomed to OSCE format. Assessors felt SimSurg had a greater risk of the scenario going “off-tangent”. Eleven themes were identified based on the GP assessor’s view of OSCEs and SimSurg. These themes and representative extracts from the surveys are presented in Table 7. 70% of respondents agreed that a reduction in the number of OSCE stations, from 12 stations to 6 stations, would still produce a valid assessment.

**Table 7:** Themes identified and example responses regarding OSCEs and SimSurg as viewed by GP assessors

**OSCE benefits** OSCEs assess specific knowledge, skills and attitudes through specific task instructions. This is not the case in SimSurg where candidates perform an overall consultation. *[OSCEs] “more specific to given clinical problem, might help in assessing GP skills that initial assessment might indicate are uncertain, or poor or indeed good.”* OSCEs can assess specific situations such as Basic Life Support, or a discussion with a colleague, which are not assessed in SimSurg. *“Benefit ought to be evidence of specific individual skills - Basic life support is a very useful test if someone says they attend lots of post-graduate training but cannot do BLS, it provides some evidence that they are not benefitting from educational activities. Talking to a practice nurse, a worried colleague, or the son of a nursing home patient are all examples of skills that are better tested in the OSCE model.”*	**SimSurg benefits** Specific skills, such as prescribing, are integrated into the consultation rather than as a separate assessment. “*GPs do not practice in bits and whole consultations are generally much more informative regarding information gathering and information sharing than OSCEs.”* SimSurg is more “authentic” to real consultations, as the scenario is based on the simulated patients’ presenting complaint. *“SimSurg is the nearest thing to real consulting, which is what GPs have to do. They are ‘real life’ situations. They cannot be ‘fixed’ as observed practice [during peer review visits] can be, with doctors suspected of inviting regular repeat patients who know what to say… The SimSurg is observed remotely which is more realistic than two or three assessors crowding into a room with the doctor and the role player.”* SimSurg is more “global”, providing a broader, more “holistic” overall picture of candidate performance. *“Simulated surgery offers a global picture of the doctors consulting which is often very valuable as some GPs have not been seen practicing for real* e.g. *if suspended. A GP can do an examination (OSCE) but when asked to integrate that into a wider whole - may perform poorly”*
**OSCE limitations** OSCEs are more intimidating as the assessor is in the room. *“I think it is more intimidating for doctor bring assessed to have assessor a in the room as opposed to watching on CCTV”* Low face validity *“…in reality we do not address specific aspects, but do so within the context of a consultation”* Penalises OSCE-naïve candidates *“Many older GPs don’t understand the concept of an OSCE so attempt a whole consultation even when they have been briefed.”*	**SimSurg limitations** Each scenario usually takes longer than a similar OSCE scenario. *“Whole consultations are needed which takes up time and may be testing similar skills repeatedly.”* Less candidate instruction means there is more potential for the consultation to go off tangent. *“In the SimSurg, the doctor may decide to defer an examination or a more detailed questioning, and the assessors may not be able to fault this decision - in the OSCE it is clearer whether or not the doctor has the relevant skill/knowledge.”* SimSurg can “mask” poor performance, as good interpersonal skills can hide candidate knowledge or competency deficiencies *“Good consulting skills/generic interpersonal skills can mask some weaknesses in knowledge or practical skills. Simulations do generally not test specific examination or practical skills or the ability to make good documentation* etc. *This is particularly a problem with training doctors who will need to have more procedural skills than experienced GPs because of hospital attachments”*

## Discussion

### Summary of findings

#### Performance assessment

Overall, GPs who were removed from the GMC register (either erased at the end of the FtP hearing or self removed during the FtP process) scored poorly compared to those that remained on the register with warnings/sanctions, as well as compared to those that remained on the register in good standing. For case examiner recommendations, GPs recommended to be erased from the register performed poorly compared to those recommended as fit to practise on a limited basis, as well as compared to those recommended as fit to practise. Further, there was significant overlap in the doctors categorised by FtP outcomes and case examiner recommendations, highlighting the consistency between the two FtP decision making stages. In terms of the demographics of doctors under investigation, these doctors tended to be male, trained outside of the UK, and obtained their medical qualification prior to 1992, consistent with doctors under investigation in prior research [12, 13].

When looking at the relationship between assessments and outcome categories, all three parts of the assessment were significantly related to outcomes of FtP investigations and case examiner recommendations, and on their own, there were no substantial differences in the predictive ability of the three components. However, there was overlap between the assessments, particularly between OSCEs and SimSurg.

#### Assessors’ opinions

Overall, assessors perceive both OSCE and SimSurg modalities to be valid and with their own merits. They thought that OSCEs allow for specific skills testing in non-consultation settings, whereas SimSurg provides an overall impression of GP ability and has higher face validity for clinical practice. Nonetheless, the assessors thought that both modalities deserved inclusion in future ToCs. However, as SimSurg assesses many of the same competencies as the OSCE, assessors thought a reduction from 12 to six OSCE scenarios is a potential modification to future GP ToC assessments.

### Implications

Our findings indicate that as stand-alone assessments, all three assessment modalities are good discriminators of various FtP investigation outcomes and case examiner recommendations, with performance in all three assessments related to final FtP ToC outcomes and case examiner recommendations in a similar manner. This is consistent with previous research demonstrating the reliability and validity of the FtP ToCs [3–5, 14].

Moreover, we found that SimSurg had additional predictive power in the presence of OSCEs and the knowledge test score for both FtP outcomes *and* case examiner recommendations. OSCEs on the other hand, did not have additional predictive power of FtP outcomes in the presence of SimSurg and the knowledge test. This is consistent with the view of GP assessors, who reported SimSurg as being the assessment modality that has the higher face validity and that provides a better overall impression of a GP’s ability.

These findings suggest that there may be an opportunity to increase the efficiency of GP ToCs by removing either the OSCE or SimSurg components. We recommend the removal of OSCEs and retaining of SimSurg in future assessments. This is because, while they were both strong predictors of FtP outcomes and case examiner recommendations, SimSurg had additional predictive value, and was reported to have higher face validity by GP assessors. This modification is further warranted as it would reduce the length of the assessment for the candidates and reduce assessor workload, while being unlikely to compromise on the validity of the GP ToCs [15].

Our findings also have wider implications for other medical assessment settings where OSCEs are used for workplace assessment, as used in many board examinations not only in the UK but also in other Western countries such as Canada and Australia. Specifically, our findings suggest that the limitations of OSCEs (including assessing individual competencies in isolation) may be reduced/overcome by the use of SimSurg, which requires an integration of multiple competencies similar to real-world medical practise. Indeed, a Canadian study examined the validity of using a hybrid simulation-OSCE assessment setting in a group of urology residents and found that the incorporation of SimSurg is likely to be a more valid assessment of trainee skills, compared to the use of OSCEs alone [16]. Further, given that many Western counties including the UK, Australia and Canada continue to rely on IMGs [17], who may not be as familiar with the OSCE format as locally trained graduates, further examination should be conducted to see whether the inclusion of simulated clinical scenarios may be a better tool to capture the skill level of such doctors compared to OSCEs. Hence, further research is needed to explore the feasibility and reliability of integration of SimSurg for such assessment purposes.

### Limitations

There are several limitations that need to be considered when interpreting the results of this study. First, due to the bespoke nature of the ToC assessments [3, 5], it is unlikely that two doctors will have taken the same test. Further, doctors that took their ToCs before October 2014 had a set roster of ten SimSurg stations. In contrast, doctors that took their ToCs after October 2014 had a bespoke SimSurg assessment tailored to their practice. Due to these factors, statistical analysis was conducted using overall scores, rather than looking at specific items or OSCE/SimSurg stations. Secondly, doctors that were erased after the FtP hearing, and those that removed themselves voluntarily during the FtP process were included in the ‘erased’ category. However, it was not known whether those voluntarily removing themselves would have been eventually erased, increasing the variability in the pool of doctors grouped as ‘no longer on the register’.

In terms of the limitations of the qualitative component, 30% of GP assessors that were asked to participate in the survey declined participation, and we were not able to ascertain reasons for this. Hence, there may be some response bias in the opinions expressed by the GP assessors who participated. Further, GP assessors were of the opinion that a reduction in OSCE stations from 12 to six would yield a valid assessment at the same time as retaining OSCE stations that tested different skills to those in SimSurg. However, it was beyond the scope of this study to determine the reliability and validity of such a modification.

## Conclusions

Notwithstanding these limitations, this study is the first to examine the utility of each GP ToC assessment modality in predicting FtP investigation outcomes concurrently with case examiner recommendations. It also provides a qualitative investigation of GP assessor’s opinion on the utility of OSCEs and SimSurg assessments. Findings from this study indicate that when examined alone, all three assessments are related with final FtP ToC outcomes and case examiner recommendations. That is, all three assessments are strong predictors of both case examiner recommendations and FtP outcomes, demonstrating their validity in the ToC assessment setting. However, our work also demonstrates that there is a lot of overlap between the assessments, particularly between the OSCEs and SimSurg. When this overlap in content is taken into account, SimSurg was a marginally better *unique* predictor of both FtP outcomes and case examiner recommendations compared to the OSCEs, although this difference was not statistically significant. This is consistent with the view of GP assessors who reported that SimSurg provides an overall impression of GP ability and has higher fidelity to clinical practice. Taken together, these findings are important in the context of increasing the efficiency and predictability of future GP ToCs and should be considered as preliminary evidence, supporting a modification in ToCs to remove the OSCE component and retain the SimSurg component. Further investigation, with consideration to factors such as time since primary medical qualification, country of primary medical qualification, and gender, are needed to further examine the effect of demographic factors on potential changes to GP ToCs in the future.

## Abbreviations

CSA: Clinical Skills Assessment
FtP: Fitness to Practise
GMC: General Medical Council
GPs: General Practitioners
IMG: International Medical Graduates
MPTS: Medical Practitioners Tribunal Service
MRCGP: Membership of the Royal College of General Practitioners
OSCE: Objective Structured Clinical Examination
SBA: Single Best Answer
SimSurg: Simulated Surgery
ToC: Tests of Competence
UCL: University College London
